# The Use of Web-Based Technologies in Health Research Participation: Qualitative Study of Consumer and Researcher Experiences

**DOI:** 10.2196/12094

**Published:** 2018-10-30

**Authors:** Patrick Cheong-Iao Pang, Shanton Chang, Karin Verspoor, Ornella Clavisi

**Affiliations:** 1 School of Computing and Information Systems The University of Melbourne Parkville Australia; 2 Health and Biomedical Informatics Centre The University of Melbourne Parkville Australia; 3 Musculoskeletal Australia Elsternwick Australia; 4 Australian and New Zealand Musculoskeletal Trials Network Melbourne Australia

**Keywords:** research subjects, consumer behavior, research design, social networking, patient portals, registries

## Abstract

**Background:**

Health consumers are often targeted for their involvement in health research including randomized controlled trials, focus groups, interviews, and surveys. However, as reported by many studies, recruitment and engagement of consumers in academic research remains challenging. In addition, there is scarce literature describing what consumers look for and want to achieve by participating in research.

**Objective:**

Understanding and responding to the needs of consumers is crucial to the success of health research projects. In this study, we aim to understand consumers’ needs and investigate the opportunities for addressing these needs with Web-based technologies, particularly in the use of Web-based research registers and social networking sites (SNSs).

**Methods:**

We undertook a qualitative approach, interviewing both consumer and medical researchers in this study. With the help from an Australian-based organization supporting people with musculoskeletal conditions, we successfully interviewed 23 consumers and 10 researchers. All interviews were transcribed and analyzed with thematic analysis methodology. Data collection was stopped after the data themes reached saturation.

**Results:**

We found that consumers perceive research as a learning opportunity and, therefore, expect high research transparency and regular updates. They also consider the sources of the information about research projects, the trust between consumers and researchers, and the mobility of consumers before participating in any research. Researchers need to be aware of such needs when designing a campaign for recruitment for their studies. On the other hand, researchers have attempted to establish a rapport with consumer participants, design research for consumers’ needs, and use technologies to reach out to consumers. A systematic approach to integrating a variety of technologies is needed.

**Conclusions:**

On the basis of the feedback from both consumers and researchers, we propose 3 future directions to use Web-based technologies for addressing consumers’ needs and engaging with consumers in health research: (1) researchers can make use of consumer registers and Web-based research portals, (2) SNSs and new media should be frequently used as an aid, and (3) new technologies should be adopted to remotely collect data and reduce administrative work for obtaining consumers’ consent.

## Introduction

### Background

Health consumers, who are users or potential users of health care services (eg, patients, families, carers, and other support people) [1], play a valuable role in health research [2,3]. They are not simply research participants these days; they are involved in research in various ways, including providing input into research design, joining advisory committees, raising public awareness, and disseminating research information [4,5]. Recruitment and engagement of consumers have been ongoing critical but challenging tasks for researchers [6-10]. A patient-centered approach has recently been advocated so that consumers can make informed decisions about whether they should participate in research [11]. However, it is often not easy to identify suitable tools to reach out to health consumers and to convey the research goals.

Recently, researchers have turned to Web-based technologies such as Web-based patient registers and social networking sites (SNSs) to recruit research participants [9,12]. Research suggests that these technologies have numerous advantages for conducting research studies, such as maintaining a list of enthusiastic potential participants [13-15], reducing recruitment cost [12,16], and providing the ability to identify hard-to-reach participants [14,17]. However, there is limited work investigating the engagement of research participants. In fact, it has been reported that there is a need to discover more clear and effective approaches for improving participant’s engagement in health research [18,19]. In addition, it is unclear whether or specifically how Web-based technologies can help with recruitment, participation, and engagement. As such, more research is needed to identify the appropriate use of Web-based technologies for these purposes.

Our review of the literature has shown that understanding and meeting the needs of health consumers can improve their experience and engagement in digital health applications [20-22] and thereby result in positive outcomes [23-25]. It is important to investigate why and how the consumers make decisions and subsequently respond to their requirements [26]. We have also identified that researchers may not fully leverage always-on Web environment and human online interactions to engage end users [27]. Given this background, we aim to investigate consumers’ needs for participating in health research and to identify opportunities to engage participants using relevant technologies.

In this study, we used a qualitative approach and obtained insights from both consumers and researchers in the area of musculoskeletal (MSK) research. We chose MSK research as our focus because many projects in this research area heavily involve consumer participants for improving their quality of life [28-31]. Therefore, this cohort can give us rich feedback about their motivations and experience about participating in research. Although our data were collected from a limited group, we expect that the insights obtained will not only be applicable to the MSK research community but also more generally to health researchers who require significant consumer involvement.

We carried out this research with the help of an Australian MSK-supporting nonprofit organization because such organizations are often considered to be an effective contact point with health consumers [32]. Through their connections, we interviewed consumers with MSK conditions for their thoughts and feedback about participating in health research as well as researchers who have conducted MSK research to understand their current practices for engaging with participants. On the basis of the interview data, we identified gaps in the current practice and proposed new directions for using technologies to improve consumer participation and engagement. We expect our study to bring a different perspective to the adoption of Web-based technologies in conducting health research, with the understanding of consumers and their needs in mind. In addition, we hope to open up a discussion about applying similar strategies to health research in other areas.

### Aims

The following 3 main research questions (RQ) comprise the aims of this study:

RQ1: What do consumers need and expect to gain from participating in health research projects?

RQ2: What current practice is used by researchers in engaging with consumers for their research projects, and what are the challenges?

RQ3: How can Web-based technologies be employed to improve engagement of research participants?

The study involves both health consumers and researchers to answer these questions. For consumers, we seek to systematically investigate their motivations for and decision-making processes about taking part in research to guide researchers to consider participants’ needs in their research design and to enhance the experience of people getting involved in academic research. On the other hand, we suggest that this study provides an opportunity for researchers to reflect on their research processes and the difficulties in engaging consumers in their studies. By comparing the needs of consumers and the current practices of researchers, we identify the strategies and opportunities to align research with participants’ expectations through the adoption of appropriate technologies.

## Methods

### Methodology

This study adopts a qualitative approach to collect and analyze data. Semistructured interviews were conducted with both consumers and researchers. In-person interviews were preferred, but phone interviews were also arranged for people who could not come on-site because of time and mobility issues. All interview sessions were conducted in the presence of authors PP and OC. This research was approved by the human research ethics committee of the University of Melbourne (Approval ID: 1648346.1).

All interviews were recorded and transcribed for data analysis. Transcripts were processed using the thematic analysis approach [33,34]. The data analysis involved reading the transcripts, mapping key ideas into codes with the open coding process, and summarizing codes into themes iteratively [35]. In the process, the number of participants connected to each theme was tracked for verifying the generality of themes. After generalizing themes, the list of themes was verified by another person in our department to assure consistency, accuracy, and quality [36].

### Recruitment

For this study, consumer participants were recruited through Musculoskeletal Australia, which is an Australian nongovernmental organization that supports people with MSK conditions nationwide. For the recruitment of other researchers in this study, we sent out invitations to their contact lists and the university’s mailing list and then reached out to the ones who responded and had previously conducted research in the area of MSK conditions. The recruitment of both consumers and researchers continued until we reached data saturation [37,38], that is, there were no new themes emerging from the collected data. Interviewees did not receive any incentives for participating in our study.

### Interview Design

For the consumer interviews, we structured our questions around their motivations, information needs, and experience of participating in research projects. In addition, the interviews focused on their views on researchers and expectations of getting involved in future research. Table 1 outlines the interview questions used in our study.

In the interviews of researchers, we started with a number of general questions to understand what research directions they were pursuing. Then, we asked about different aspects of their research projects, ranging from recruitment, research methods, and difficulties, to costs and incentives. In addition, we investigated the channels and the types of media used for promoting their research. Table 2 shows the interview questions for researchers.

### Participants

Representing consumers, we completed interviews with 23 people having MSK conditions. The mean age of the participants was 51 years (SD 15.7 years, range 15-72 years). About half of our participants (48%, 11/23) reported that they had previously taken part in academic research. Table 3 summarizes the demographics of our participants.

**Table 1:** The list of interview questions for consumers.

Category	Questions
Demographic	Age, gender, remoteness, work status, and conditions
Motivation	What makes you want to find out more about academic research? Why is a research project of interest to you? What do you expect to gain from participating in a research project?
Opportunities to get involved in research	Where do you get information about particular research projects? How do you find research that is relevant to you? What websites or tools do you use to find research? What are your search criteria?
Experience	What is your overall experience with getting involved in a research study? What are the difficulties and challenges? How do you think technologies can improve your participation?

**Table 2:** The list of interview questions for researchers.

Category	Questions
General information	What kinds of studies do you normally carry out? Will the participants need to be involved for a long term? What is the typical time commitment required from them?
Recruitment	What are the difficulties you face when recruiting participants for your research? What are the factors that lead to a successful recruitment? What information do you provide to recruit potential participants? How do you screen relevant or appropriate participants? How much time do you spend on recruitment? What incentives do you give to your participants? How much cost do you spend on marketing?
Channels	What channel do you currently use to recruit? How do you think that technologies can help to improve the participant recruitment and engagement in health research? Would you able to use materials such as images, photos, or videos to recruit participants? Why or why not? Have you considered using a consumer register or a potential participant database for recruitment?

**Table 3:** Demographics of consumer participants in our study (n=23).

Demographics		n (%)
**Gender**		
	Male	5 (22)
	Female	18 (78)
**Living area**		
	Metro	14 (61)
	Rural	9 (39)
**Work status**		
	Full-time	6 (26)
	Part-time	5 (22)
	Unemployed	12 (52)

Representing researchers, we interviewed 10 researchers who study various aspects related to MSK conditions. These included clinical research in back pain and spinal pain, the quality of life with osteoarthritis, observational studies after surgery, as well as epidemiology research. All researchers were based in Australia and possessed a minimum academic level B (Lecturer; equivalent to Assistant Professor in the US system) position. They played the role of chief investigator of academic research projects and directly led ongoing or past studies in the MSK research area.

## Results

### Overview

This section presents the data collected from the interviews with both consumers and researchers. We further organize the results into 2 subsections: consumer needs and researcher strategies.

### Consumer Needs

This section reports on the consumers’ needs (*consumer needs, CN*), which can be further broken down into 4 subthemes: research as learning opportunities (CN1), research transparency and updates (CN2), trustworthiness (CN3), and mobility (CN4). The corresponding consumer identifier is listed after each quote.

#### CN1: Research as Learning

Many of our consumer participants, particularly those with less access to information sources, viewed researchers as being on the frontier of science, and, therefore, they hoped to learn something from research. Participants reported that they were enthusiastic about taking part in research because they would have a chance to enquire about the latest remedies and treatments that might be useful to them. In addition, research was a learning opportunity for them to obtain new knowledge about their conditions:

For me it is the possibility of new information on effective treatments becoming available to me and I do a lot of my own looking around but—or just trying to keep on top of new developments I guess.

C4

Research is one of the things that people want to know about because it gives them a sense as a set of hope[s] that things might be different for them in the future.

C5

#### CN2: Research Transparency and Updates

Consumers indicated that receiving more clear information about the purpose, the scope, and the protocols of a research project is essential for them to get involved. As people with MSK conditions sometimes found participating in research activities difficult in terms of access or mentally demanding, they tended to choose carefully beforehand and only invested in research that was relevant and beneficial to them:

[Researchers need to] be explicit about what they expect from participants. Because, as I said, there’s a lot of anxiety with a lot of people with chronic illness about how much they have to give to anything energy wise...

C5

On the other hand, consumers wanted to receive feedback regarding the progress of the study as well as the final results. Furthermore, they were disappointed when they did not receive any communication regarding research that they had participated in. As noted by our interviewees, this appears to be a very common issue:

In the end, I didn’t ever get to hear any results back. I’ve got obviously, thanked for my participation, but it was disappointing that I never heard anything back.

C21

#### CN3: Information Sources and Trustworthiness

A large portion of our consumers suggested that they would not proactively search for relevant research to participate in. Instead, it is essential for someone to inform them about eligible studies. Many of our participants highlighted the importance of health professionals such as general practitioners, rheumatologists, and physiotherapists for introducing or actively referring them to research projects. One participant said, “I probably don’t go about looking for something until I hear about something” (C1).

In addition, the participants reported that the trustworthiness of information sources was critical for them to consider regarding whether the research is worthwhile to participate in. Some people emphasized that they would not take part in any pharmaceutical or marketing research. They also had concerns about how to find genuine research on the internet, for example, from Google. In this case, community services, support groups, and health professionals played an important role to provide information to potential research participants:

I’d probably go by word of mouth and ask other people or other clinicians or something like that rather than just go randomly onto Google.

C3

I’d rather someone I trust to tell me where to go [for research]...

C7

#### CN4: Mobility and Rural Locations

In the consultations with our participants, we understood that people with MSK conditions needed extra considerations for their conditions and special needs in the research design. For instance, some older participants suggested that they preferred physical contact and building a social connection with the researchers. They tended to talk more and to have more interactions in the progress. These factors need to be considered before adopting the use of technologies:

I think for some of the older-older people prefer to have a face to face conversation. They like to talk to people, they like that option of being able to have a chat.

C4

Besides, a significant ratio of the consumers had low mobility, which limited their ability to travel for academic studies. This was a particular problem for people living in rural areas and made them feel isolated. As a result, technology aids were suggested to address these limitations and to make research more accessible. One participant expressed, “I’ve got to weigh up the time and the effort involved physically and how much pain it would cost me... the online stuff is fine” (C8).

### Researcher Strategies

This section presents the strategies used by researchers (*researcher strategies, RS*) to assist with recruitment and to maintain consumers’ engagement for their research. Overall, 4 main strategies have been identified: establishing rapport with consumers (RS1), handling changes in research (RS2), using technologies (RS3), and designing research for the participants (RS4). The corresponding researcher identifier is shown after each selective quote.

#### RS1: Establishing Rapport With Consumers

Researchers pointed out the importance of establishing a connection with consumers at the beginning of the recruitment to engage them with the research project. Although many MSK research projects recruited through clinicians and health professionals, their interaction with the potential participants was crucial:

If that person [the clinician] who has sort of clinical responsibility for the patient is into it and is sort of an advocate for the research, then I think that’s what—in some ways the recruitment lives or dies by that. That person is really sort of going into bat for your study...

R8

It was also helpful to involve consumers in the very early stages of the research, even in the design of the trial, so that they could be informed about the purpose of the research and the benefits to them. It was worthwhile for consumers to learn more about the research and the rationale behind it:

[The] number one is just engaging the consumer right at the beginning, so that we understand that we’re answering and addressing the question that’s of real importance to consumers, even in the design of the trial...trying to work out how to explain to consumers why it’s so important and it is a good investment of their time.

R5

Some researchers found that the time spent by research team members to talk and build up a relationship with the potential participants was a good investment in some studies:

That person [the staff member] will take the time to have a chat with them about the project, and I think when you establish the rapport like that...I think if you had that connection they are more likely to consent to being part of the study.

R2

Conversely, poor communication would endanger the recruitment. This also highlights the needs of researchers and facilitators to manage the relationship with consumers:

We have had circumstances where the communication between the research assistant and the participant wasn’t great, and so then participants, they get disappointed and they decided to drop the study...The studies that we’ve been most successful in keeping patients in the study, are studies where we actually have someone actually spending time talking to patients and that particular person is someone who develops a relationship with the participants.

R1

With these challenges in recruiting participants by the researchers themselves, they used patient groups or consumer organizations to help with the recruitment. These organizations had direct connections to the consumers. As a researcher suggested, “Going through a consumer organization or a professional body is probably a better way to go” (R2).

#### RS2: Handling Changes in Research

Changes are inevitable in research. Researchers found that it was a challenge to go back to the participants to request more information or to obtain consent when there were changes in their research:

If you just list one very specific question, then if you’re going to re-analyze that data with different questions, or if you need to go back to the twins [the participants of that study] and re-interview them, then you need to go back [sic] and ask for permission again.

R1

Some researchers reported on their experience of obtaining multilevel consent, which allowed consumers to choose the level of involvement such that they were comfortable throughout the research. In this way, the experience of the participants was enhanced, and the administrative burdens of obtaining additional consents were lowered:

I suppose we're talking about really, the consent process here. So in a way, what would be best practice would be multi-level consent. So, you could have the tick all box, which is, “I’m happy to be contacted by anybody about anything at any point,” and everything backwards from there, up to, “Don’t contact me about anything, I’ll contact you,” and everything in between. And, in a way, I suppose offering that spectrum is the most appropriate thing to do and, in some ways, supporting people to make an informed decision about where they want to sit on that spectrum.

R6

#### RS3: The Use of Technologies

Researchers agreed that media such as images and videos were more powerful. As such, they started to use SNSs such as Facebook and YouTube to help promote their research. Text information can carry a large amount of precise information, but often it is not sufficiently engaging. In contrast, short videos could help to deliver the basic message and the background of the research. However, researchers also mentioned that they did not know where to obtain appropriate images or how to modify the images for the best results on SNSs. For videos, there were also reports on having difficulties with editing videos and producing satisfactory output quality:

We’re very much happy with technology that still involves images, for example, so Skype and tele-rehabilitation is a good example, patients and participants tend to be quite happy with that. Technologies that don’t involve images, or such as just text and email, and that sort of thing, I think, they tend to be a problem.

R1

One of my concerns with it that there is so little (video and audio)—it’s all written information. Like there’s no other way of receiving that information. And video and audio and all that sort of stuff is so useful.

R3

Well, why don’t we start a three-minute video placed on YouTube, explaining what it is...And, in under 12 months, we’ve had about 26,000 views of that video.

R6

The first of these things are videos, like, YouTube videos, to express things you are researching, using a language that is more easy to understand and provide a bit of background about the condition or the things you are working on.

R2

#### RS4: Designing Research for the Participants

Time commitment was one of the issues identified by MSK researchers in their research. The time of the participants was precious, and the researchers tried to minimize the time required to travel for data collection. Some researchers used Web-based recruitment tools and survey platforms. However, for electronic data collection, it was important that surveys be kept to a reasonable length to avoid participants dropping out in the middle of a lengthy session. Such details in the research design directly affected the dropout rate of participants:

So, in terms of collecting the data, we try to keep this data collection session as quick as we can, anything over one hour becomes a problem...if it’s a survey, an electronic survey, it needs to be much less than that, it needs to be no longer than 20 minutes, otherwise it becomes a major issue, the participants tend to drop out of the study.

R1

In addition to time commitment, travel distance was reported as another issue that affected participation. People living in remote areas had more difficulties gaining access to studies. Researchers described the use of Web-based tools to help these people to participate in research and to collect data:

We had a lot of rural and even remote people that we had. We did virtual focus groups, so an online focus group, and we had the face to face ones with people living in metro.

R2

Another challenge pointed out by our interviewees was that arranging meetings and focus groups for participants was difficult. Participants had different schedules and might not have a common time to meet. This was particularly true for focus group research because it required a minimum number of people to be present:

In order to hold a focus group, you have to have between six to eight minimum participants who can come on the same day at the same location at the same time. So, it was a process of asking people if they could give me [sic] of their availability and then trying to match a general availability with a specific case didn’t always work out. Yeah, so getting participants with similar availability was the main complication for focus group research.

R7

## Discussion

### Principal Findings

Through the interviews, we have gained insight into the motivations and the needs of consumers who participate in research projects. We have also learned that researchers have already identified some of the key issues and are adopting strategies to keep consumers engaged. For example, the following points were noted:

- Researchers establish a good rapport with participants, which further fosters more learning opportunities for research participants (RS1→CN1).
- Researchers invest the effort to build an ongoing relationship with consumers from the beginning of the recruitment, which helps to create trust and confidence in consumers’ hearts (RS1→CN3).
- Researchers visit and request new consent again when there are changes in their research, which allows greater transparency and updates relevant to participants (RS2→CN2).
- Researchers have taken up new technologies to help consumers who live in rural locations or have low mobility participate in the research (RS3→CN4).
- Researchers have started to design their research to be better tailored for the potential participants, such as using online focus groups, splitting meetings, and reducing the time required for taking a session (RS4→CN4).

**Figure 1:**
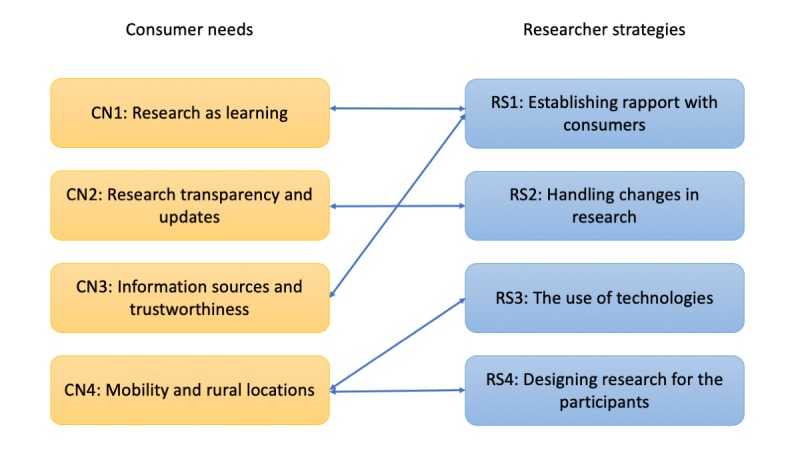
Key consumer needs and researcher strategies identified through the research.

**Figure 2:**
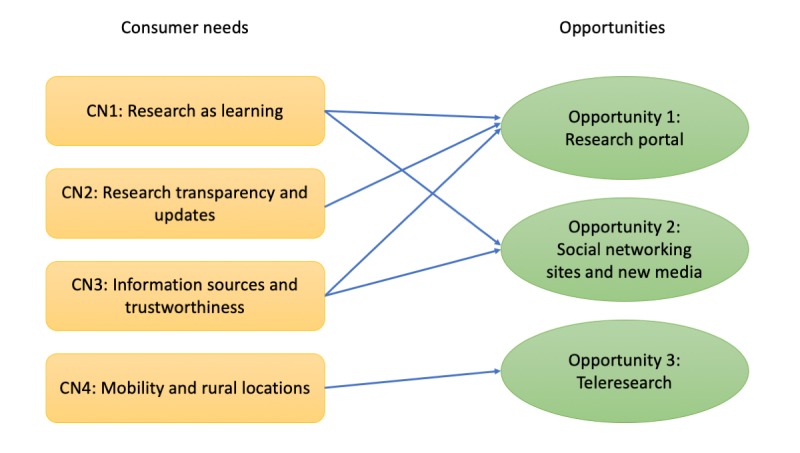
Opportunities for supporting consumer needs with Web-based technologies.

Figure 1 summarizes consumer needs and how these needs are being addressed by current researcher strategies. Although effort has been devoted to engage consumers in research activities, we identify 3 areas that can address their needs better through the use of Web-based technologies (Figure 2). These will be elaborated in the later parts of this section.

#### Opportunity 1: Research Portal

Past research has shown that patient registers can effectively recruit participants [9,10,13,39,40]. Taking a step further, we propose that such registers can be augmented with streamlined research portals that further address different consumer needs. A consumer-facing research portal can provide multiple functions, including promoting research opportunities, allowing electronic participant recruitment, and sending notifications and updates to relevant users through the Web.

As suggested by our results, consumers tend to have passive information-seeking behavior [41,42] for acquiring information about health research. In other words, people do not realize the needs of getting information and instead rely on other people to trigger them to start seeking information. As a result, they do not notice that research opportunities actually exist unless they get informed. In this case, a research portal can store contact information of consumers and enable researchers to notify them of new studies. This will ignite their interest to learn more about the new research and consider participation (CN3).

In addition, a research portal can show a list of research projects and present the current status, updates, and even recent publishable results of such projects. This approach can improve research transparency and provide timely updates to consumers (CN2). This can fulfill their needs for getting news and updates at their convenience and eventually help them to learn more about the study. A research portal can be seen as another credible resource for consumers, which allows them to understand and acquire the latest knowledge about their conditions and thereby addresses the learning needs (CN1).

On the other hand, consumer organizations can help with engaging participants in health research [43]. First, consumer groups can help to advocate the use of patient registers and research portals as well as to disseminate the research information. Also told by researchers (RS1), working with consumer groups is helpful for their research, which results in establishing rapport and trust with consumers (CN3). Besides, the information on consumer-faced health portals often requires a higher level of literacy [44,45] as there are disparities of the literacy and the knowledge levels between authors (such as researchers and clinicians) and consumers. We anticipate that research portals might have similar issues. In this case, consumer organizations can play an intermediate role to review research material and interpret the content in an easy-to-understand manner for consumers. This will facilitate the learning, understanding, and involvement in health research (CN1).

Furthermore, a research portal can act as a credible information source, which helps to mitigate the issues of confidence and trustworthiness (CN3). A Web-based research portal can make the identity of the project owners and the administrators more transparent so that visitors can know of the nature and the ownership of the portal (eg, *is it noncommercial?* or *is it supported by a pharmaceutical company?*). The branding of the portals can also help to build up the confidence and eventually improve consumer satisfaction [46]. Finally, a centralized-managed research portal can be easier managed and secured by information and technology professionals who have the expertise in the operational and cybersecurity perspectives that health researchers do not normally have.

#### Opportunity 2: Social Networking Sites and New Media

As reported by researchers (RS3), SNSs are being used to carry out recruitment, which is consistent with other literature [47-49]. Despite the wide use of SNSs, it remains a challenge to recruit research participants properly through these sources. It is reported that extra consideration of recruitment design as well as technical work are needed to prevent repeated attempts from the same person and biased samples when research is advertised on these social networking platforms [48,49]. Additionally, SNS users are not evenly distributed across different age levels and ethnicity background [50]. Therefore, we suggest that the use of SNSs should be only a part of the whole recruitment, unless the targeted cohort is specifically people who use SNSs heavily.

The recent trend of using multimedia content on SNSs brings new opportunities to researchers. Researchers understand that using images and video clips has advantages over the classical text-based material. Recent papers have pointed out that posts with rich content (eg, images and videos) capture more attention on SNSs [51-53], which researchers can leverage to promote their research more effectively. On the other hand, videos often provide a shorter and clearer presentation of information and can employ visual aids to help watchers to learn and understand the research context (CN1). The use of human faces in videos helps to build a relationship with the audience (RS1). This gives a starting point to establish the trust (CN3) and the rapport (RS1) with the potential participants.

However, the use of new media on SNSs creates new technical burdens for researchers. As highlighted in the results (RS3), researchers found it difficult to use or manipulate multimedia on SNSs. This aligns with previous literature that using SNSs for health research is resource-demanding [12,54]. To overcome the issue, we suggest a *social media kit* can be designed for researchers to provide sources of properly licensed (eg, licensed under Creative Commons that allows the reuse of materials) images and videos that are technically usable on SNSs. In addition, more instructions and tutorials can be offered for effectively editing and using new media on SNSs. Universities and institutions can consider setting up a team of social media specialists for helping researchers to broadcast their research on the internet with low overheads and matching the audience with a propensity for relevant research topics.

#### Opportunity 3: Teleresearch

With the growing momentum of faster internet networks and mobile and wearable technologies, researchers can consider using the latest development of teleresearch technologies to collect data and conduct research activities. For example, mobile apps can be used to log user activities and could be an alternative to conducting diary studies; videoconferencing software (eg, Skype and Zoom) can be used as a channel for group interviews and focus groups. Recent work about wearable gadgets such as smart socks [55] allows researchers to collect body measurements remotely. These technologies provide participant-friendly solutions for diverse needs of time, place, and mobility (CN4).

Additionally, technologies can assist with the change in the research process (RS2). The latest research suggests that using dynamic consent to collect consent digitally can help consumers to make decisions for their participation in research and reduce the administrative burden of researchers [56,57]. For instance, dynamic consent can split the entire consent of a study into a few consent items, and users will only be prompted to give consent on the fly when relevant experiments are performed. As such, the system can notify the participants to provide additional permissions online without the need to contact them by physical means. This simplifies the complexity on the researcher side handling changes in the research. In addition, participants can choose to transfer their digital consent to third parties, which enables easier collaborations and data reuse across research teams. Recent trials have started to explore the deployment of digital consent and its efficacy in various settings [58].

### Limitations

We acknowledge several limitations of our study. Both the samples of consumers and researchers may not represent the entire population because of the relatively small number of subjects. For consumers, the sample was biased in favor of women and older cohorts. However, this can be explained by the fact that more women and older people suffer from MSK problems [59]. In addition, the recruitment of this research was conducted through a single consumer organization, which might affect the diversity of the sample. Finally, the adoption of technologies may impact the participant cohort with lower digital and health literacy. More research in the future should investigate the impact on this cohort.

### Conclusions

On the basis of the interviews with consumers and researchers, we summarize 4 major types of consumer needs as well as 4 strategies used by researchers for engaging participants. On the basis of these findings, we argue that 3 areas of Web-based technologies can be employed to assist in addressing consumer needs and engaging with research participants: research portals, SNSs, and teleresearch. Additionally, our research outcomes lead to a better understanding of human participants and offer an opportunity to reflect on the research design. The analysis presented in this paper is not just relevant to a single discipline but is also applicable to other types of health research.

## Abbreviations

CN: consumer needs
MSK: musculoskeletal
RS: research strategies
RQ: research question
SNSs: social networking sites
